# Elevated serum neuropeptide Y levels are associated with carotid plaque formation in Chinese adults: a cross-sectional study

**DOI:** 10.1186/s12872-023-03660-7

**Published:** 2023-12-12

**Authors:** Wan-da Wang, Hui-li Lin, Yan-li Zheng, Sheng-nan Wang, Yao-guo Wang

**Affiliations:** Department of Cardiology, the Second Affiliated Hospital of Fujian Medical University, Quanzhou, Fujian Province China

**Keywords:** Carotid plaque, Atherosclerosis, Neuropeptide Y, TNF-α

## Abstract

**Background:**

Carotid plaque (CP) formation is an important consequence of atherosclerosis and leads to significant complications. Levels of neuropeptide Y (NPY), which is a sympathetic neurotransmitter, are elevated in cardiovascular diseases. It also has important roles in inflammatory conditions. This study aimed to explore the relationship between serum NPY and CP and to study further the influence of NPY and inflammatory factors on CP.

**Methods:**

This cross-sectional study was conducted among 300 adults who underwent a health examination at the Second Affiliated Hospital of Fujian Medical University in Fujian Province, of whom 177 were finally enrolled. The participants were divided into the CP (n = 120) and non-CP (NCP) or control (n = 57) groups according to the results of carotid artery color Doppler ultrasound. The CP group was further classified into stable plaque (SP, n = 80) and vulnerable plaque (VP, n = 40) groups based on plaque characteristics. Serum NPY and pro-inflammatory cytokine tumor necrosis factor-α (TNF-α) levels were examined. Univariate and correlation analyses were used to evaluate the correlation between serum NPY levels, pro-inflammatory cytokines, and the CP phenotype.

**Results:**

The serum NPY and TNF-α levels of patients in the CP group were significantly higher than those in individuals from the NCP group [ (177.30 ± 43.29) pg.mL^− 1^ vs. (121.53 ± 40.16)pg.mL^− 1^, P < 0.001; (41.94 ± 14.19) pg.mL^− 1^ vs.(33.54 ± 13.37)pg.mL^− 1^, P = 0.003]. The serum NPY levels of the patients in the VP group were significantly higher than those in patients from the SP group [(191.67 ± 39.87)ng.L^− 1^ vs.(170.12 ± 43.37)ng.L^− 1^, P = 0.01, P < 0.05]. Serum TNF-α and NPY levels were positively correlated among patients from the CP group (r = 0.184, P = 0.044). The binary logistic regression analysis showed that serum NPY and TNF-α were independent influencing factors of CP [(OR = 1.029, P < 0.001);(OR = 1.030, P = 0.023)]. The area under the ROC curve of NPY predicting the CP showed statistical significance at a value of 0.819.

**Conclusion:**

Together, elevated serum NPY levels seem to be associated with the occurrence of coronary atherosclerosis in Chinese adults.

## Background

Atherosclerosis (AS) is the primary pathological basis of most cardiovascular diseases and is characterized by chronic inflammatory damage caused by endothelial dysfunction, which usually affects multiple arteries [1]. Atherosclerotic cardiovascular disease is a major public health concern and a primary cause of death worldwide [2]. Inflammation-related cells are essential components of AS plaques. Inflammatory reactions cause dysfunction of the carotid artery endothelial cells and increase the production of intercellular adhesion molecules by promoting macrophage phagocytosis and transforming them into foam cells. When AS develops, unstable AS plaques rupture, possibly causing platelet aggregation and thrombosis and inducing acute cardiovascular events, such as vascular stenosis or occlusion [3]. The carotid artery is more prone to AS owing to its unique “Y-shaped” bifurcation and impact from high pressure due to blood flow. Carotid AS (CAS) induces systemic AS [4]. Therefore, early assessment of the occurrence of CAS and carotid plaque (CP) stability could significantly aid in preventing adverse cardiovascular events.

Neuropeptide Y (NPY) is an abundant sympathetic neurotransmitter distributed throughout the central and peripheral regions of the body [5]. Serum NPY is released from the sympathetic postganglionic fibers, adrenal medulla, and platelets [6]. However, elevated plasma NPY levels have been documented in diseases, such as hypertension [7], chronic heart failure [8], and renal failure [9], wherein sympathetic outflow is increased. NPY can regulate inflammatory responses, vascular endothelial function, and oxidative stress and promote platelet activation by binding to G protein-coupled receptors [10]. Local injections of NPY into the carotid artery of rats also promote the occurrence of AS lesions and vascular occlusion after carotid angioplasty [11].

Therefore, as an active neuroendocrine regulatory product, NPY may be significantly associated with AS. However, there is little real-world data on the relationship between NPY and CP, which is necessary to determine effective interventions and strategies in this regard. This study aimed to investigate whether serum NPY levels are related to the progression of CP lesions and to further explore the correlation between serum NPY levels and inflammatory responses.

## Materials and methods

### Study type

This was a cross-sectional study.

### Research participants

Between June 2020 and January 2022, 300 participants who underwent color Doppler examination of the carotid artery at the Second Affiliated Hospital of Fujian Medical University, China were screened. All participants signed an informed consent form. This study was approved by the Ethics Committee of our hospital.

The major inclusion criteria were as follows: (1) age, 18–75 years, (2) patients who underwent color Doppler examination of the carotid artery at a designated time in this hospital, and (3) patients who were conscious and could co-operate until study completion.

The exclusion criteria were as follows: (1) cancer, hematological disease, organic heart disease, autoimmune disease, hyperthyroidism, Cushing syndrome, hyperparathyroidism, infection, history of myocardial infarction and stroke, history of tuberculosis, chronic alcohol abuse, pregnancy, lactation, diabetic foot, diabetic ketoacidosis, or hyperglycemic hyperosmolar state; (2) diseases causing abnormal NPY levels, such as psychiatric disorders and epilepsy; (3) participants with unqualified specimens (e.g., hemolysis); and (4) missing information on anthropometric data and metabolic components.

Finally, 120 participants were included in the CP group (n = 120). Based on the plaque characteristics, the CP group was further divided into stable plaque (SP, n = 40) and vulnerable plaque (VP, n = 80) groups. Patients without CP were included in the control group (NCP group, n = 57).

### Data collection

The anthropometric data collected primarily included sex, age, smoking history, height, and weight. Systolic blood pressure (SBP) and diastolic blood pressure (DBP) were measured with the participants in seated and resting positions.

All blood samples were collected at 8 am after fasting for 10–12 h and centrifuged at 3,000 × *g* for 5 min to separate the serum. One part of the supernatant was immediately assessed for routine clinical biochemical indicators using a fully automated biochemical analyzer (Beckman LX20, USA) in the Laboratory Medicine Department of our hospital, and the other part of the supernatant was stored at -80 °C for enzyme-linked immunosorbent assay (ELISA). Serum NPY and tumor necrosis factor-α (TNF-α) levels were measured using specific ELISA with an intra-assay error of < 10% and an inter-assay error of < 12%.

### Carotid artery ultrasonography

Carotid artery ultrasound was performed on all participants using a color Doppler ultrasound diagnostic instrument (LOGIQ S7 Expert) at a transducer frequency of 4–9 MHz by two experienced physicians blinded to the patients’ conditions. Using the Mannheim criteria, CP was described as a focal zone invading the arterial lumen by at least 0.5 mm, > 50% of the surrounding intima-media thickness values, or a thickness of 1.5 mm above the distance between the lumen-intima and the media-adventitia interfaces [12]. If plaques were found, further evaluation of plaque stability was carried out. Plaques with smooth surfaces, intact intima, and high internal echogenicity, were defined as stable plaques while those with fewer smooth surfaces, incomplete inner membranes, and internal low echo, isoechoic, and mixed echo plaques, were defined as unstable plaques. If the ultrasound results were inconclusive, two senior physicians, expert at color ultrasound, reached a consensus after discussion.

### Statistical analyses

All data were statistically analyzed using SPSS v26.0. We reported patient characteristics using means and standard deviations for normally distributed variables, medians and interquartile ranges (IQR) for non-normally distributed variables, and proportions for categorical variables. Independent samples t-test, Mann–Whitney U test, and χ^2^ test were used for comparison of normally distributed continuous, non-normally distributed continuous, and categorical variables, respectively. Spearman’s correlation coefficient was performed to evaluate the relationship of NPY with TNF-α. A binary logistic regression analysis was performed to determine the association between serum NPY levels and CP. ROC curves were drawn to evaluate the effect of NPY in predicting CP. A two-tailed p < 0.05 indicated statistical significance.

## Results

### Comparison of baseline data

The age showed a significant difference between the CP and normal groups (P = 0.001). No significant differences were observed between the CP and normal groups with respect to sex, smoking, hypertension, diabetes, body mass index (BMI), and levels of UA, SBP, DBP, MAP, total cholesterol (TC), triglycerides (TG), low-density lipoprotein (LDL-C), high-density lipoprotein (HDL-C), or FPG (P > 0.05) (Table 1).

**Table 1:** Baseline characteristics of the study participants (N = 177)

Characteristic	NCP group	CP group	P
n	57	120	
Sex			0.458
Male	27	64	
Female	30	56	
Smoking	8(14.04%)	26(21.67%)	0.228
Hypertension	21(36.84%)	54(45%)	0.305
Diabetes	19(33.33%)	36(30%)	0.723
Age(y, x ± s)	53.14 ± 9.33	58.07 ± 8.77	0.001
BMI(kg.m^− 2^, x ± s)	26.06 ± 4.19	25.55 ± 4.00	0.436
UA(umol.L^− 1^, x ± s)	338.40 ± 116.62	369.69 ± 121.93	0.108
SBP/mmHg	126.0(115.00-139.00)	132.5(124.00-151.75)	0.146
DBP/mmHg	78.00(71.00-89.50)	83.00(75.25–89.75)	0.095
MAP/mmHg	96.33(86.83-105.67)	99.1(92.42-110.58)	0.258
TC/mmol.L^− 1^	4.53(3.79–5.27)	4.64(3.75–5.66)	0.787
TG/mmol.L^− 1^	1.35(0.88–2.01)	1.46(1.16–1.98)	0.554
LDLC/mmol.L^− 1^	2.64(2.10–3.51)	2.91(2.06–3.66)	0.361
HDLC/mmol.L^− 1^	1.12(0.96–1.30)	1.08(0.84–1.38)	0.424
FPG/mmol.L^− 1^	5.64(4.63–7.20)	5.63(5.00-7.23)	0.959

Data were reported as the mean (SD) or median (IQR: Q1-Q3). The t-test or Mann-Whitney U test or χ^2^ test was used for comparisons between NCP group and CP group

BMI, body mass index; UC, uric acid; SBP, systolic blood pressure; DBP, diastolic blood pressure; MAP, mean arterial pressure; TC, total cholesterol; TG, triglyceride; LDLC, low-density lipoprotein cholesterol; HDLC, high-density lipoprotein cholestero; FPG, fasting plasma glucose

### Comparison of NPY and TNF-α levels in CP conditions

Serum NPY and TNF-α levels were higher in the CP group than in the NCP group(P < 0.05) (Table 2). Specifically, however, the NPY levels in the SP group were significantly lower than those in the VP group (P < 0.05) (Table 3).

**Table 2:** Comparison of serum NPY and TNF-α levels between the NCP group and the CP group

Item	NCP group	CP group	P
n	57	120	
NPY/ng.L-1	121.53 ± 40.16	177.30 ± 43.29	< 0.001
TNF-α/pg.mL-1	33.54 ± 13.37	41.94 ± 14.19	< 0.001

**Table 3:** Comparison of serum NPY and TNF-α levels between the SP group and the VP group

Item	SP group	VP group	P
n	80	40	
NPY/ng.L^− 1^	170.12 ± 43.37	191.67 ± 39.87	*P* = 0.010
TNF-α/pg.mL-1	41.13 ± 14.65	43.57 ± 13.26	P = 0.376

### Correlation between NPY and TNF-α in the CP group

**Fig. 1:**
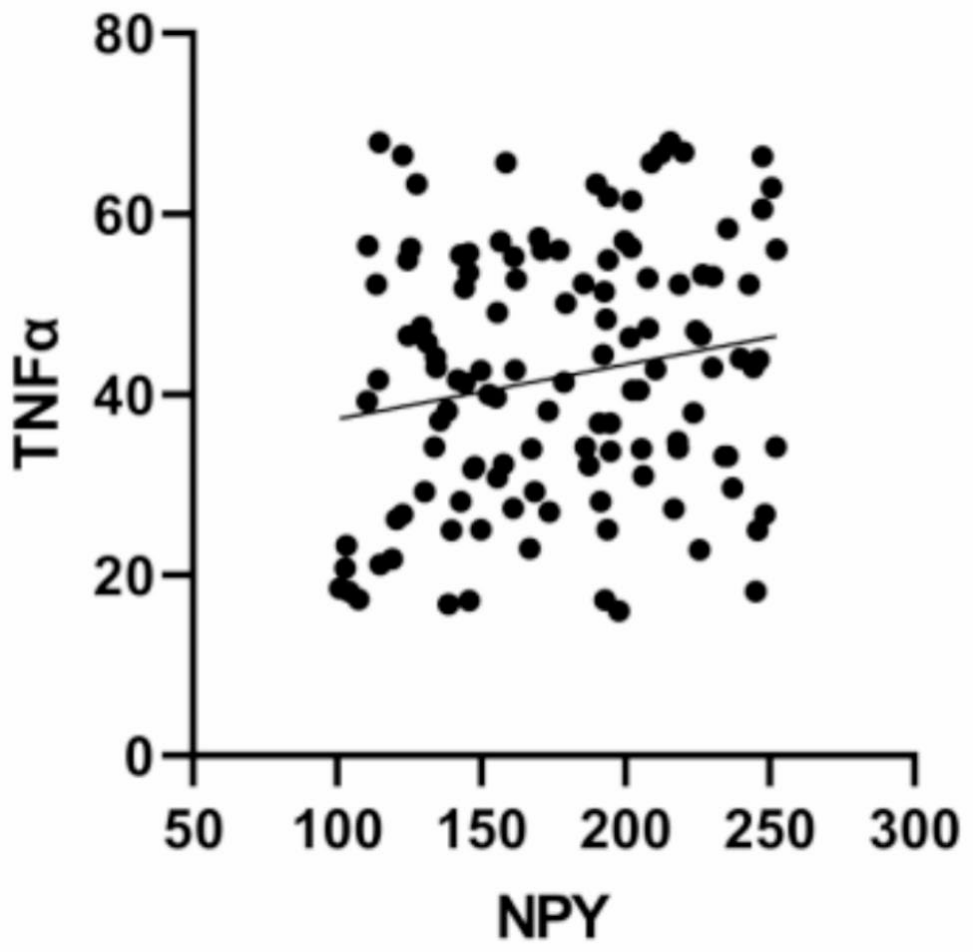
The correlation analysis between NPY and TNF-α in the CP group

Serum NPY levels were positively correlated to TNF-α levels in the CP group (r = 0.184, P = 0.044) (Fig. 1).

### Correlation between NPY and TNF-α levels and CP

We performed bivariate regression analysis to analyze the specific correlation between serum NPY and TNF-α levels and CP. Correlations were measured using odds ratios. After adjusting for age and TNF-α, higher levels of NPY remained independently associated with CP (OR = 1.029, P < 0.001). After adjusting for age and NPY, TNF-α was an independent influencing factor for CP (OR = 1.030, P = 0.023)(Table 4).

**Table 4:** Binary logistic regression for CP

Variable	Binary logistic regression					
	B	Sex	Wald	OR	95%CI	*P*
Age	0.027	0.025	1.717	1.027	0.984–1.086	0.263
NPY	0.029	0.006	19.564	1.029	1.016–1.041	< 0.001
TNF-α	0.030	0.016	3.097	1.030	0.997–1.062	0.023

### NPY predicts CP

**Fig. 2:**
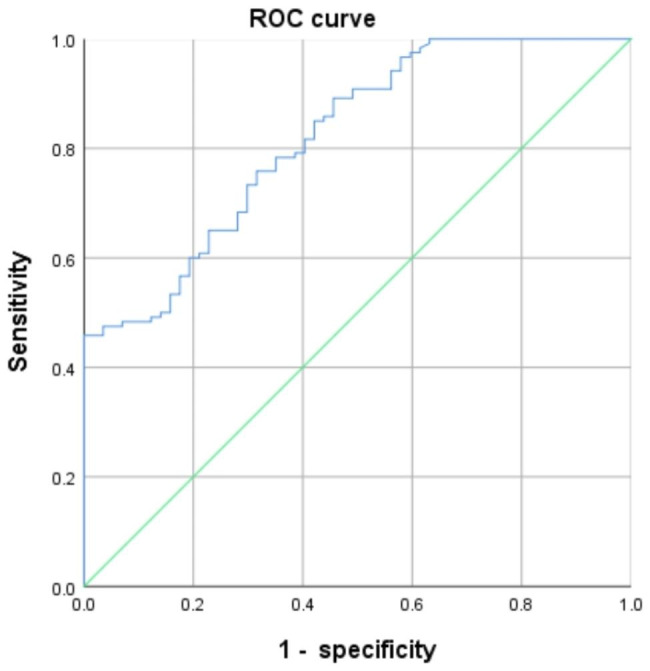
The ROC curve

To evaluate the predictive value of NPY for the occurrence of CP, the ROC curve was mapped, where NPY was used as a test variable and CP was assigned as a state variable (0 for NCP, 1 for CP). NPY had a good predictive value for the occurrence of CP (AUC = 0.819; 95% CI, 0.756–0.882; σ = 0.032; P < 0.001; Fig. 2). The optimal cutoff value for NPY is 189.60 ng.L^− 1^.

## **Discussion**

CP is a well-known imaging biomarker for predicting recurrent vascular events. Carotid artery ultrasound is increasingly being used to assess the activity, quality, and morphology of plaques. Herr et al. suggested that increased carotid intima-media thickness was associated with an increased risk of cardiovascular events [13]. Moreover, the echogenicity and fragility of the CP have been considered independent predictors of coronary artery events [14]. Exploring the relationship between serum NPY levels and CP may provide a potential biomarker for assessing the risk of cardiovascular events and a perspective on the onset of AS.

Our results showed that serum NPY levels were higher in the CP group than in the NCP group. After adjusting for factors, such as age, blood lipids, and TNF-α, serum NPY levels independently correlated with the occurrence of CP. Previous clinical studies have suggested that NPY concentration is an independent predictor of coronary heart disease and ischemic stroke [15]. Wu et al. showed that downregulation of NPY and its receptor expression in animals had an anti-atherosclerotic effect [16]. This is consistent with our findings, which suggest that NPY may be associated with the occurrence of CP.

In addition, the serum NPY levels of participants in the VP group were higher than those of individuals in the SP group. Previous studies have shown that NPY activates the extracellular signal-regulated protein kinase (ERK1/2) pathway in mice through the Y1 receptor, thereby inducing the expression of matrix metalloproteinase 8 in macrophages in a dose-dependent manner [17], hydrolyzing the extracellular matrix, and disrupting plaque stability. NPY binds to the Y2 receptor on endothelial cells, thereby promoting the secretion of nitric oxide and vascular endothelial growth factor by these cells within these plaques and inducing angiogenesis [18]. New blood vessels in plaques exacerbate plaque growth and increase the risk of bleeding and rupture, causing thrombosis. The areas with myocardial infarction, myocardial cell apoptosis, and expression and activity of caspase-3 were significantly reduced in NPY-knockout rats, and cardiac systolic function was significantly improved [19]. These findings suggest that, in the presence of CAS, NPY could promote unstable lesions in patients with CP.

Similarly, our results showed that the peripheral blood TNF-α levels of patients in the CP group were higher than those in the patients in the NCP group. This suggests that the inflammatory levels in blood vessels in patients with CP may significantly increase. TNF-α is a mononuclear factor primarily produced by monocytes and macrophages. It upregulates the expression of adhesion molecules and inflammatory cytokines and induces an AS inflammatory response [20]. Inhibiting TNF-α can reduce the progression of AS in ApoE^−/−^ mice [21]. We found a positive correlation between NPY and TNF-α levels, thereby implying a possible correlation between NPY and a significantly elevated inflammatory response in the CP group. Inflammatory reactions can gradually increase lipid core volume, decrease extracellular matrix components, and cause thinning of plaque fiber caps, thereby increasing plaque fragility [22], which may explain the high levels of NPY in the VP group.

However, no significant differences were found in the serum TNF-α levels between the SP and VP groups (P = 0.376). This may be related to the time of procuring the serum TNF-α samples from patients with VP. The degree of inflammation may vary at different stages of AS development [23]. During the process of plaque formation and rupture, we could not accurately determine the timing of the most severe inflammation. Therefore, predicting the optimal time point for the peak release of serum TNF-α levels in CAS participants was challenging. Whether the serum TNF-α levels differ between the SP and VP groups requires further studies.

This study had some limitations. First, the participants in this study were primarily from the Fujian province; therefore, the conclusion is primarily applicable to the southern China population and partially to those in its other parts, such as northern and western China. Second, this study did not identify a concentration range within which serum NPY exerts its biological functions and did not investigate the number of and functional changes in NPY receptors. Thirdly, participants with chronic diseases regularly administer medications under the guidance of specialized physicians, but there are significant differences in the duration of administration, type, dosage, and manufacturer of these medications. Therefore, we did not include specific medication information in the analysis of the results. Finally, this was a cross-sectional study, and we could not conclude that elevated serum NPY levels can promote the development of CAS in Chinese adults.

NPY can regulate inflammatory responses, vascular endothelial function, and oxidative stress; promote platelet activation by binding to G protein-coupled receptors; activate the ERK1/2 pathway in mice through the Y1 receptor, inducing the expression of matrix metalloproteinase 8 in macrophages in a dose-dependent manner [17]; hydrolyze the extracellular matrix and disrupt the stability of plaques; and bind to the Y2 receptor on endothelial cells, promoting the secretion of NO and vascular endothelial growth factor by endothelial cells in plaques, and promoting angiogenesis [24]. New blood vessels in AS not only exacerbate the growth of plaques, but also increase the risk of bleeding and rupture, leading to thrombosis. The study by Pankajakshan et al. revealed that NPY-Y2 mRNA expression was 10 times greater in the smooth muscle cells of symptomatic plaque than that in healthy carotid artery, and the TNF-α-stimulated smooth muscle cells of healthy carotid artery increased the transcription of NPY-Y2 mRNA [25]. Accordingly, these mechanisms suggest that in the presence of CAS, NPY is likely to promote unstable plaques in patients with CAS.

NPY exerts its biological effects by binding to receptors, specifically the inflammation related NPY-Y1, NPY-Y2, and NPY-Y5 receptors [26]. The high distribution of these receptors in the hypothalamic tissue suggests that exploring the correlation between NPY levels in the brain, peripheral inflammatory response, and CAS may be a valuable approach. In addition, NPY regulates immune responses in diverse, complex, and contradicting manners [27]. Exploring the regulatory mechanisms of NPY on innate/adaptive immunity may provide novel insights and effective interventions for the management of AS.

## Conclusions

This study suggests that higher serum NPY levels are independently associated with CP and vulnerability to CP, possibly owing to the promotion of inflammation by NPY.

## Data Availability

All data generated or analysed during this study are included in this published article.

## Abbreviations

NPY: Neuropeptide Y
CP: carotid plaque
NCP: non-carotid plaque
CAS: Carotid atherosclerosis
AS: atherosclerosis
ELISA: enzyme-linked immunosorbent assay
